# Extracorporeal Off-Pump Antegrade Cerebral Perfusion in Reconstructive Surgery for Type A Aortic Dissection With Cerebral Malperfusion

**DOI:** 10.7759/cureus.71549

**Published:** 2024-10-15

**Authors:** Marina Elias, Abubakar I. Sidik, Vladimir Mironenko, Sergey Garmanov, Maxim L Khavandeev, Abdulmajid Ilyas Mohammad Shafii

**Affiliations:** 1 Department of Cardiothoracic Surgery, RUDN University, Moscow, RUS; 2 Department of Cardiothoracic Surgery, Bakulev Scientific Center for Cardiovascular Surgery, Moscow, RUS; 3 Department of Cardiothoracic Surgery, V.K. Gusak Institute of Emergency and Reconstructive Surgery, Donetsk, RUS; 4 Department of Cardiovascular Medicine, RUDN University, Moscow, RUS

**Keywords:** aorta, aortic arch, branch-first technique, cerebral blood perfusion, cerebral malperfusiion, dissection, extracorporal antegrade cerebral perfusion, total aortic arch replacement, type a acute aortic dissection

## Abstract

Introduction

Type A aortic dissection (TAAD) is a life-threatening condition that often leads to cerebral malperfusion (CM), a severe complication that can result in permanent neurological damage. Traditionally, a cardiopulmonary bypass (CPB) with selective antegrade cerebral perfusion (ACP) is employed during aortic arch reconstruction to protect cerebral circulation. However, the use of CPB carries inherent higher risks, including embolic events, hypothermia, and interrupted cerebral perfusion, especially in patients with CM. This study evaluates an innovative off-pump extra-corporeal ACP technique using an axillo-axillary shunt to provide uninterrupted bihemispheric cerebral perfusion during branch-first stage total aortic arch replacement (BF-TAR) for TAAD with CM; the shunt depends on cardiac contradiction to transfuse blood from the donor axillary artery to the recipient axillary artery, which then flows to through the carotid and vertebral arteries to the brain.

Methods

Between 2021 and 2023, 18 patients with TAAD complicated by CM underwent BF-TAR; the novel axillo-axillary shunt technique was employed for ACP because of the risks of ischemic neurologic injury. Outcomes measured included operative mortality, neurological complications, cardiopulmonary bypass times (measured after completion of the branch-first stage), and overall morbidity.

Results

The axillo-axillary shunt provided stable, continuous ACP in all patients. No new permanent neurological deficits were observed. Five (27.8%) patients experienced transient neurological symptoms such as blurred vision, dizziness, and confusion, which resolved within 48 hours. Operative mortality was 5.6% (1 patient), and minor complications included transitory lower limbs ischemia in 3 patients (16.7%) and deep sternal wound infection in 1 patient (5.6%). All transitory complications were managed by “watchful waiting”. The mean CPB time was 145.3 ± 48.6 minutes, while the mean cross-clamp time was 100.6 ± 17.4 minutes, which was better than the average of 227 ± 32 minutes and 147 ± 23 minutes reported in other studies. Postoperative imaging confirmed well-reconstructed aortic arches with no residual malperfusion or graft-related complications.

Conclusion

The off-pump axillo-axillary shunt technique provides a safe and effective method for maintaining continuous bihemispheric cerebral perfusion during total aortic arch replacement in patients with TAAD complicated by CM. This approach minimizes the risks associated with CPB, including embolic events and interrupted cerebral perfusion while achieving favorable neurological and surgical outcomes. Further studies with larger cohorts and longer follow-ups are warranted to validate the long-term benefits of this innovative technique.

## Introduction

Aortic dissection is a life-threatening condition with a mortality rate of 1%-2% per hour during the first 48 hours; it requires immediate surgical intervention, particularly when cerebral malperfusion (CM) is present [1]. Type A aortic dissection (TAAD), which involves the ascending aorta, can lead to devastating neurological outcomes if blood flow to the brain is compromised during the acute phase of dissection [1]. The management of these cases presents a significant challenge for cardiac surgeons due to the dual need to restore aortic integrity and maintain adequate cerebral perfusion to prevent neurological damage during surgery [2,3].

Traditionally, cardiopulmonary bypass (CPB) with hypothermic circulatory arrest has been employed to protect the brain during aortic dissection repair. Also, a separate head pump maybe used to provide cerebral blood whilst the reconstruction of the arch branches takes place. However, these methods carry inherent risks, including embolic events, prolonged operative times, and increased chances of neurological deficits postoperatively [4,5]. Moreover, the use of hypothermia, absence of pulsatile cerebral blood flow, altered perfusion velocity, and periods of unilateral antegrade cerebral perfusion (ACP) associated with these methods all contribute to the disruption of cerebral autoregulation, potentially exacerbating neurological deficits [6]. Also, the unihemispheric ACP in the use of CPB is often interrupted during critical stages of the procedure, further increasing the risk of ischemic brain injury [6].

The drawbacks of CPB for ACP are particularly severe in cases of TAAD complicated by CM, resulting from thrombosis and/or extension of the dissection into the aortic arch branches (AAB). In these scenarios, restoring adequate ACP becomes more complex and critical [7]. In the study by M. Ghoreishi evaluating the risk factors for the development of neurological complications during surgical treatment of acute TAAD, it was shown that prolonged CPB and selective on-pump ACP increase the risk of stroke [7].

The branch-first technique of aortic arch replacement using a trifurcation graft simplifies the delivery of continuous ACP, and takes advantage of intracranial and extracranial anastomoses of carotid artery branches to augment contralateral cerebral perfusion if the circle of Willis is complete; that is, there are functioning anastomoses between the left and right cerebral arteries [8,9,10]. We developed a new technique of ACP for the branch-first stage of the procedure that involves the use of an extra-corporal axillo-axillary shunt. This technique is pump-independent (off-pump), as it allows the transfusion of blood from the left (donor) to the right (recipient) axillary artery. This off-pump technique ensures uninterrupted bihemispheric ACP during the branch-first stage, which involves the resection of the aortic arch branches (AAB) and their reconnection to branches of a trifurcated graft [6]. By using the patient’s own circulation to maintain ACP, the method reduces the complexity and risks associated with pump-assisted procedures.

The off-pump extracorporeal ACP offers a way to maintain cerebral blood flow without the use of CPB, avoiding some of the complications associated with traditional approaches. Early studies have shown promising results, with reduced rates of cerebral injury and better neurological outcomes in high-risk patients [6].

In this article, we describe the use of our off-pump technique ACP in patients with TAAD complicated by CM. We provide a detailed overview of the surgical method, discuss its advantages and challenges, and present the outcomes of patients who underwent this innovative procedure.

## Materials and methods

Patient selection

Between 2021 and 2023, 18 patients underwent branch-first total aortic arch replacement for TAAD complicated by thrombosis and/or dissection extending into the AAB. The exclusion criteria were patients under 18 years of age, significant stenosis of the left subclavian artery (as this could hinder the left axillary artery's effectiveness as the donor artery), and patients in cardiogenic shock, as it would impair cardiac contractility needed for cerebral perfusion. Seven patients had acute dissection while the remaining 11 patients presented with chronic aortic dissection. All patients were initially managed with blood pressure, heart rate, and pain control. All patients displayed moderate to severe manifestations of CM at the time of surgery, with symptoms including chest pain, dyspnea, generalized weakness, dizziness, headache, amnesia, and nausea. The mean age of the cohort was 47 ± 14 years, and 11 of the patients were men. All patients had hypertension, 4 (22.2%) had diabetes mellitus, and 2 (11.1%) had atrial fibrillation. Due to the high risk of irreversible cerebral ischemic injury, associated with the involvement of the AAB, an alternative perfusion strategy was required to ensure adequate cerebral protection during the procedure.

Preoperative assessment

To evaluate the extent of the dissection and the involvement of the AAB, all patients underwent ultrasonography and multislice computed tomography (CT) with contrast enhancement of the aortic arch branches. Contrast-enhanced CT revealed that all patients with TAAD had minor leakages between the false and true lumen of the aortic arch. In 11 out of 18 patients, the dissection affected all branches of the aortic arch, while in 7 patients, the dissection was limited to the innominate artery, with the true lumen compressed by the false lumen.

The functional completeness of the circle of Willis was assessed using the Matas test, which involved compressing the common carotid artery while measuring blood flow velocity in the ipsilateral middle cerebral artery through transcranial Dopplerography; this revealed a functionally incomplete circle of Willis in all patients. The Dopplerography data showed a decrease in systolic blood flow velocity in one or both middle cerebral arteries by more than 50% from the baseline level during compression of the ipsilateral common carotid artery. This finding was a requirement for the application of an extra-corporal off-pump axillo-axillary shunt to ensure uninterrupted bihemispheric ACP throughout the surgical procedure.

Surgical technique

Axillo-Axillary Shunt for Continuous Cerebral Perfusion

**Figure 1:**
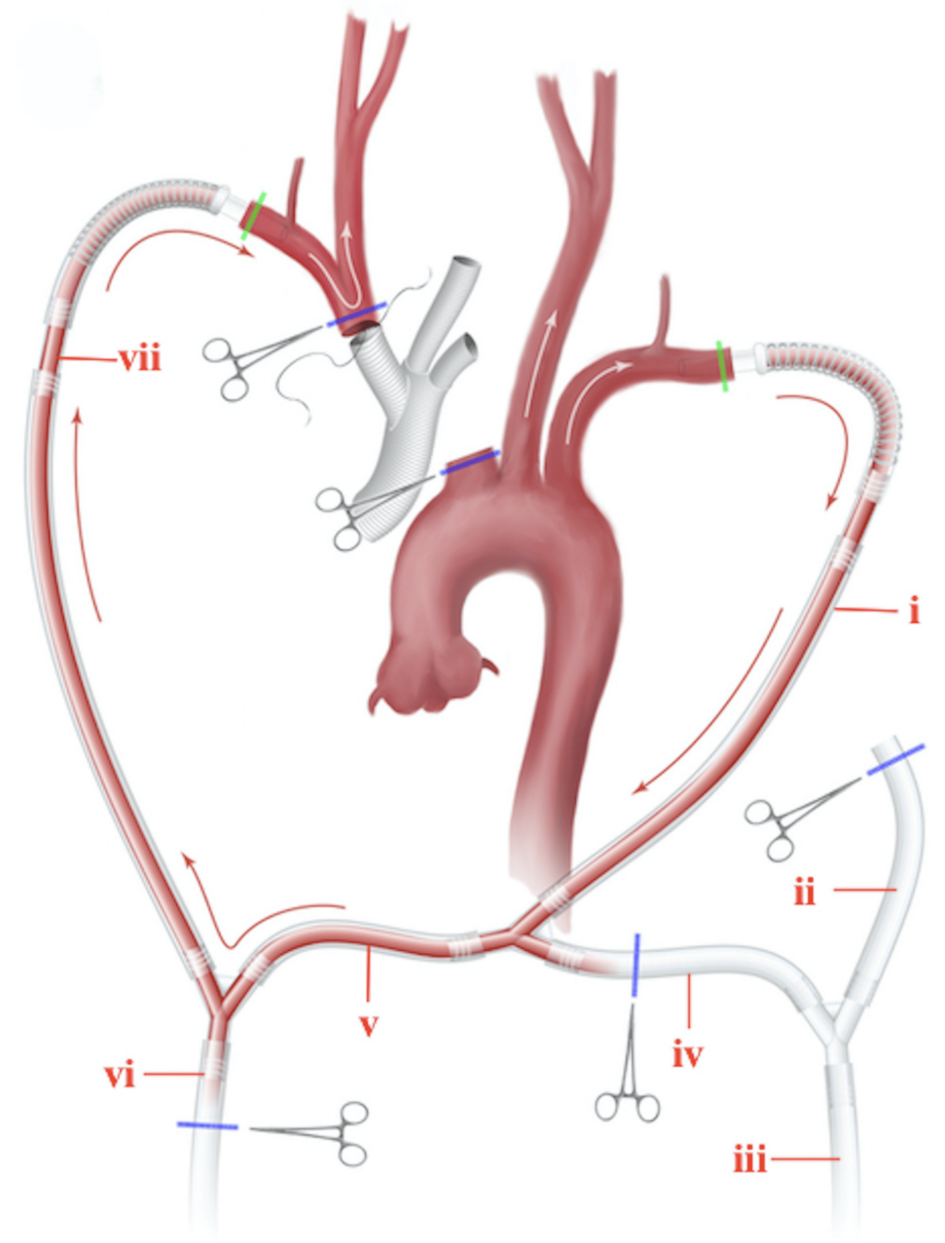
Circuit depicting bihemispheric cerebral perfusion along the off-pump axillo-axillary shunt (i). Cannula to LAA, which is also connected to both the CPB and head pumps. It operates in off-pump mode during branch-first stages; (ii). Cannula from the CPB pump to be connected to the aortic tube graft to augment systemic circulation after distal aortic anastomosis; (iii). Arterial cannula to the CPB pump; (iv). Cannula connecting the CPB pump to the perfusion circuit; (v). Cannula from the head pump to the circuit; (vi). Arterial cannula to the head pump; (vii). Cannula from the heat pump to RAA. LAA: left axillary artery; RAA: right axillary artery; CPB: cardiopulmonary bypass Image credit: The authors

Given the potential for irreversible cerebral ischemia should the AAB be cross-clamped for extended durations during the branch-first stage, an axillo-axillary shunt is created using cannulae within a perfusion circuit to ensure uninterrupted bihemispheric ACP via the carotid and vertebral arteries. The shunt operates in off-pump mode throughout the entire branch-first phase. However, if cardiac contraction becomes inadequate to sustain cerebral blood flow, as indicated by low oxygen saturation levels, cerebral perfusion pressure below 55 mmHg, and a flow velocity of 600 ml/min, it can be switched to on-pump mode using CPB or a head pump (Figure 1).

Perfusion Circuit

To create the perfusion circuit, the axillary arteries are exposed in the infraclavicular fossa and mobilized. After median sternotomy, access is gained to the ascending aorta, aortic arch, and its branches, as well as the proximal portion of the descending aorta for the distal anastomosis. To minimize the risk of atheroembolization, careful handling of the aorta and its branches is crucial. A systematic approach, beginning with the innominate artery, improves visibility for each following branch, as the previously addressed vessel can be retracted, creating more space and enhancing the arch's mobility for the procedure. The mean size of the aortic root was 55 ± 5 mm, the ascending aorta 80 ± 6 mm, and the aortic arch 45 ± 3 mm.

Heparin (600 U/kg) is administered, and both the right and left axillary arteries (RAA and LAA) are cannulated and connected to each other using intermediary cannulae to form the axillo-axillary shunt. The shunt is then connected to the CPB and the cardioplegia pump (acting as the standby head pump for selective ACP) using additional cannulae and connectors; this completes the perfusion circuit setup. The inferior and superior vena cavae are also cannulated, and the left heart is vented through the right superior pulmonary vein. At this stage, although the shunt and the perfusion circuit as a whole are fully set up, they are not in operation (the cannulae to the axillary arteries are clamped and the CPB pump is off at this stage).

Aortic Arch Reconstruction

**Figure 2:**
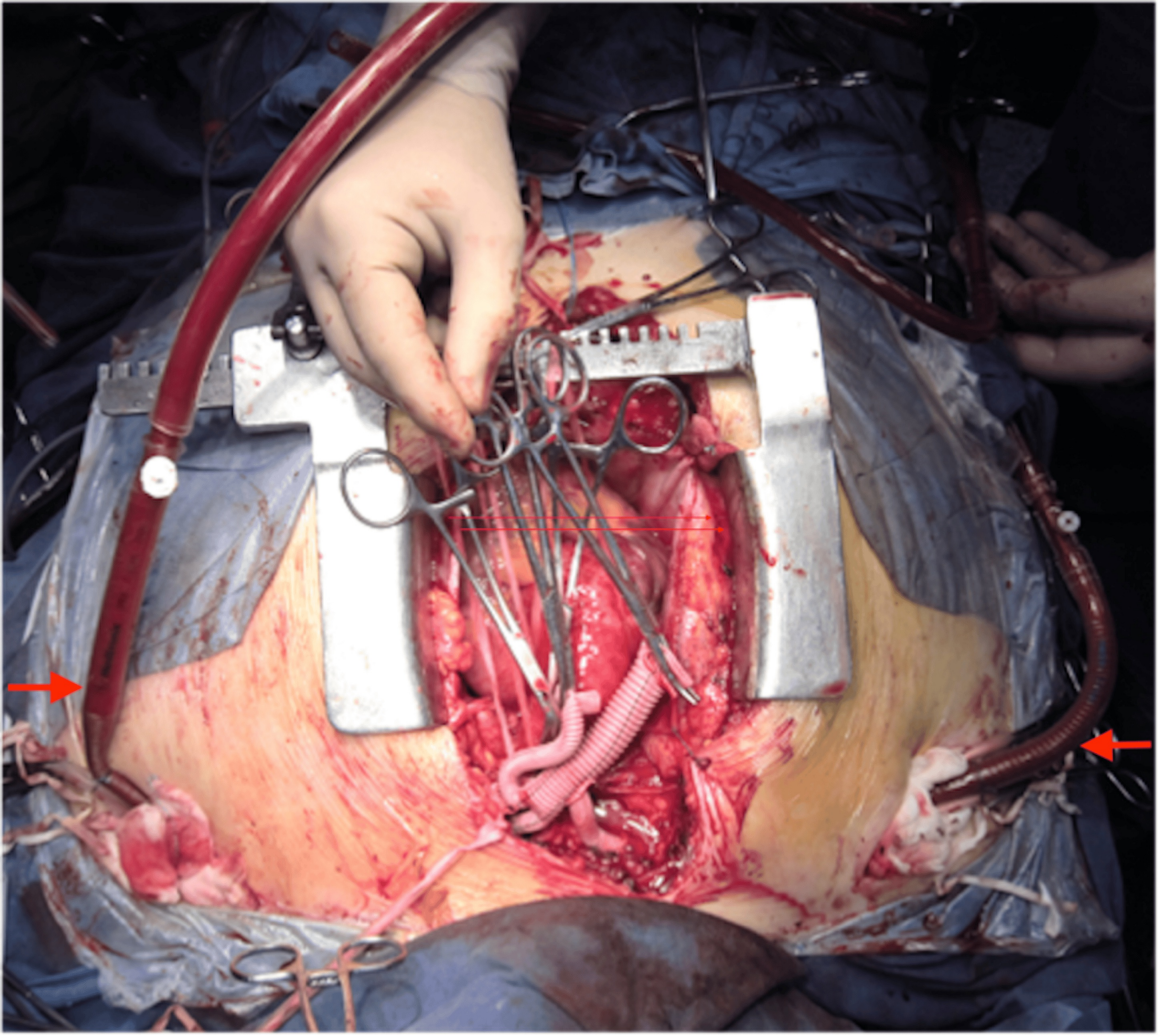
Resection and anastomosis of the aortic arch branches to the trifurcated graft in a sequential manner The red arrows show the cannulae to axillary arteries forming the axillo-axillary shunt.

To begin the branch-first stage, the innominate artery is cross-clamped and the cannulae to the axillary arteries are de-clamped to become operational (blood flows from the LAA to the RAA). Then, the AAB are sequentially cross-clamped proximal to the bifurcation and distal to the origin from the aortic arch and divided; dissected areas are resected, thrombotic debris is removed by suction and with forceps, and the AAB are anastomosed using 4-0 or 5-0 polypropylene sutures in an end-to-end fashion to an appropriately sized trifurcated graft (Bakulev Scientific Center of Cardiovascular Surgery, Moscow, Russia), starting with the innominate artery and then the left carotid artery (Figure 2). The shunt ensures ACP in the off-pump mode throughout the branch-first stage, by transfusing blood from the LAA to the RAA and subsequently to the right subclavian artery, innominate artery, and common carotid arteries. During this stage, blood flow along the carotid arteries is maintained at a mean pressure of 55 mmHg and a velocity of 600 ml/min, which are our preferred values for normal cerebral perfusion.

Perfusion Circuit and On-Pump Phase

**Figure 3:**
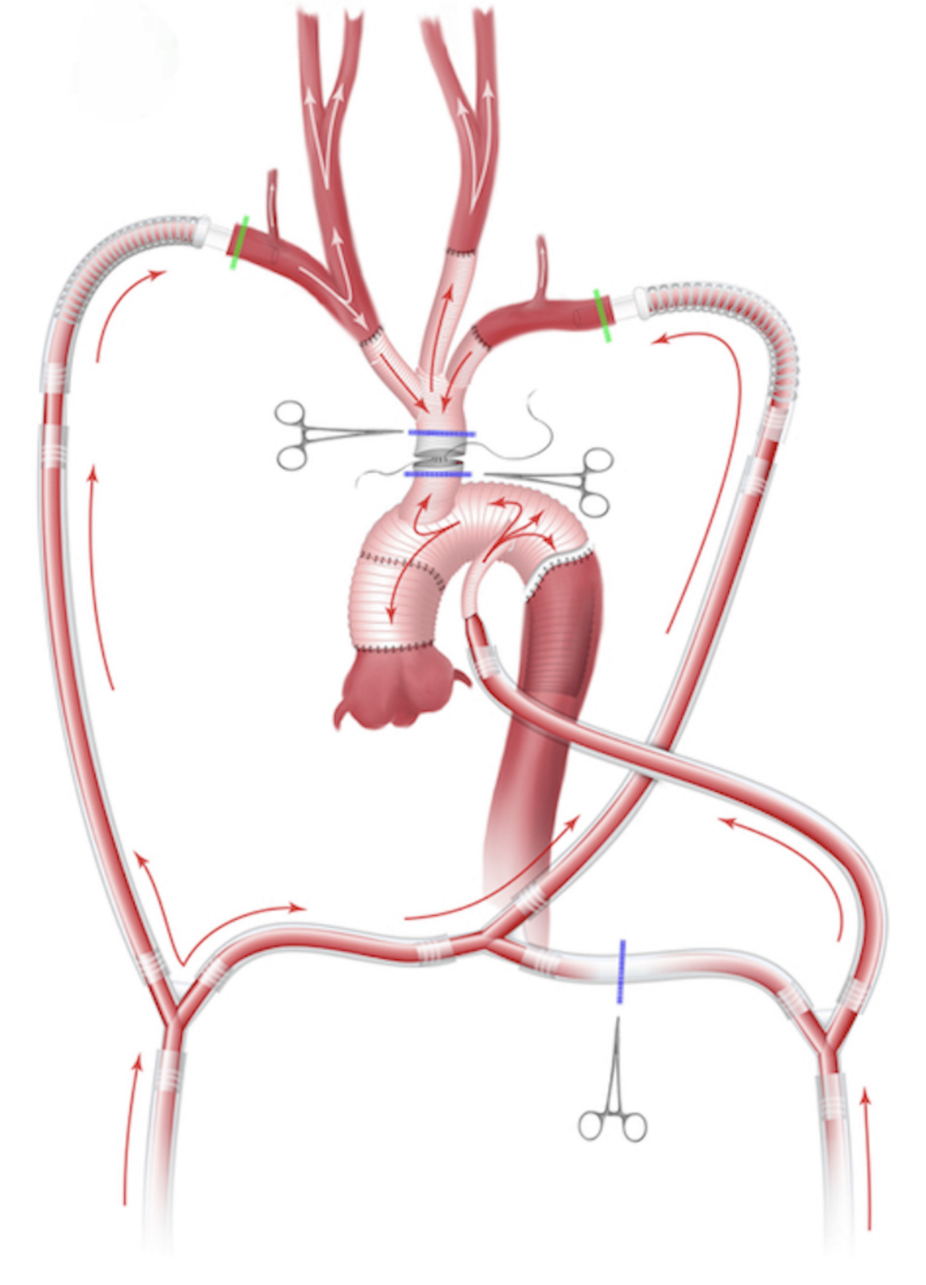
Completed branch-first total aortic arch replacement The common stem of the trifurcated graft is anastomosed in an end-to-side to the tube graft. The side port of the tube graft is used for systemic circulation while the resection and anastomosis of the left subclavian artery take place. Image credit: The authors

After the branch-first stage, CPB is started in the on-pump mode (the entire circuit setup becomes operational), and the patient is cooled to 26°C for the reconstruction of the ascending aorta and aortic arch. Supracoronary ascending aortic replacement was performed in 13 patients and Bentall’s operation in 5 patients who had concomitant aortic valve failure; the frozen elephant trunk procedure was incorporated into 11 of the procedure’s due to dissection extension into the descending aorta. During the proximal anastomosis of the graft to the aortic root, CPB provides systemic blood supply through the LAA and RAA. After the distal aortic anastomosis was completed using the Basex tube graft (Bakulev Scientific Center of Cardiovascular Surgery, Moscow, Russia), the left subclavian artery was resected anastomosed to the third branch of the trifurcated graft, and the common stem of the graft is passed under the innominate vein and anastomosed to the aortic graft in an end-to-side fashion (Figure 3).

Monitoring and Postoperative Assessment

**Figure 4:**
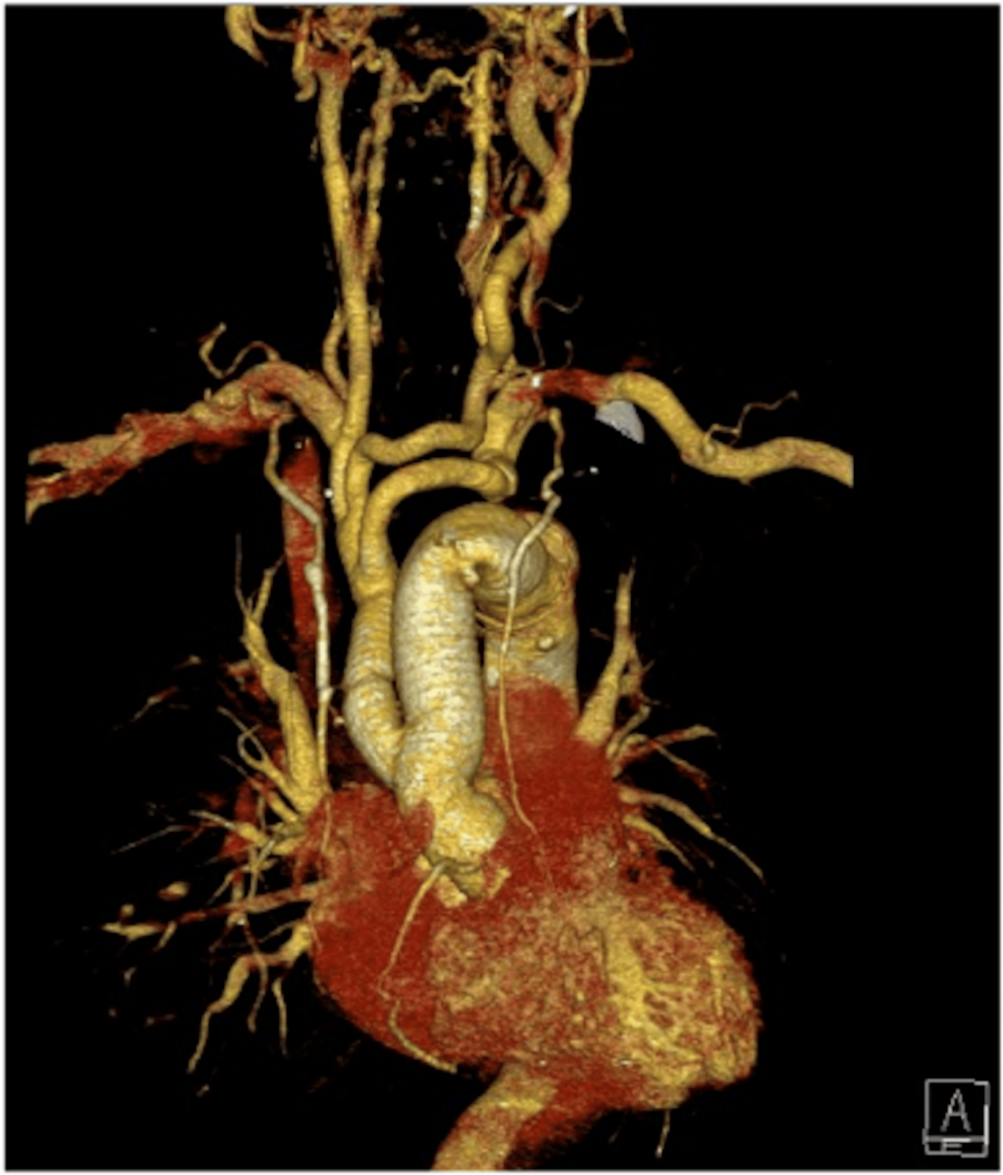
Postoperative CT angiography showing a complete repair of the aortic dissection using the branch-first technique

This perfusion technique ensures continuous bilateral ACP along the carotid and vertebral arteries throughout the surgery. Cerebral perfusion is monitored using cerebral oximetry, electroencephalogram, bispectral index (BIS) monitoring, and transcranial Doppler. Throughout all stages of the surgery, the hemoglobin oxygen saturation level (rSO2), monitored via cerebral oximetry, stayed within the normal range of 60-80%, with no occurrences of critically low rSO2 values (below 40%). Aortic regurgitation on intra-operative and postoperative TEE was negligible after the procedure. A follow-up CT angiography showed a well-reconstructed and functioning ascending aorta, aortic arch, and branches (Figure 4).

Completion of Procedure

Following the completion of the reconstructive procedure, ACP is rechecked to confirm adequacy. Once verified, the axillary arteries are decannulated, the patient is weaned off CPB, hemostasis is ensured at the cannulation sites, and the wounds are closed in layers.

End Points

The primary outcomes assessed included operative mortality and major complications such as reoperations for any cardiac-related causes, renal failure, deep sternal wound infections, extended ventilation or intubation periods, and cerebrovascular events (permanent strokes). Secondary outcomes included intraoperative measurements like the CPB, myocardial ischemic time, lower-body circulatory arrest, and ACP times. The ACP time is defined as the moment when both systemic and cerebral perfusion were restored through a single arterial inflow, which coincided with the completion of the proximal anastomosis of the trifurcated graft. Postoperative follow-up was conducted annually at the aortic clinic through office visits and CT scans. After discharge, patients were also managed by their cardiologist, who reported improved quality of life and normal echocardiographic findings. No patients were lost to follow-up.

## Results

Neurological outcomes

Continuous monitoring of cerebral oxygenation during the procedure and early postoperative period demonstrated stable rSO2 values throughout. There were no new permanent neurological deficits or strokes in any of the patients. Five (27.8%) patients experienced transient neurological symptoms, including mild confusion, reduced vision, and dysarthria, which resolved within 48 hours postoperatively. These neurological symptoms were not severe enough to justify invasive tests like MRI (Table 1).

**Table 1:** Operative outcomes ACP: antegrade cerebral perfusion; AAS: axillo-axillary shunt; CPB: cardiopulmonary time

Intraoperative variables	Values
Mean total ACP time, min	150 ± 30.8
Mean off-pump ACP time (with AAS), min	34.2 ± 12.2
Mean on-pump ACP, min	109.5 ± 26.6
Mean total CPB time, min	145.3 ± 48.6
Mean aortic cross-clamp time, min	100.6 ± 17.4
Mean circulatory arrest time	40.3 ± 15.8
Mean myocardial ischemic time, min	94.2 ± 28.1
Mean total mechanical lung ventilation, hrs	15.4 ± 5.0
Postoperative outcomes	Values
Operative mortality, n (%)	1 (5.6)
Permanent stroke, n (%)	0 (0.0)
Transient ischemic attack, n (%)	5 (27.8)
Re-operation for repair failure, n (%)	0 (0.0)
Transitory real failure, n (%)	3 (16.7)
Transitory lower extremities ischemia, n (%)	3 (16.7)
Deep sternal wound infection, n (%)	1 (5.6)

Mortality and morbidity

There was 1 (5.6%) in-hospital death that occurred on postoperative day five due to cardiogenic shock. Minor complications included superficial wound infections in 2 patients (11.1 %), both of whom were managed with antibiotics. Deep sternal wound infection was noticed in 1 (5.6%) patient with previously poorly controlled diabetes mellitus. No patients required reoperation or prolonged mechanical ventilation (greater than 21 days of mechanical ventilation for at least six hours per day). The mean hospital stay was 12 ± 2 days. All patients were discharged in stable condition with no significant residual neurological or cardiovascular deficits (Table 1).

Postoperative imaging

Postoperative CT angiography performed prior to discharge revealed well-reconstructed and patent aortic arches and branches in all patients. There were no instances of graft-related complications such as thrombus formation or occlusion. All patients demonstrated normal cerebral perfusion postoperatively, with no signs of residual CM or ischemic brain injury.

## Discussion

The present study demonstrates the efficacy and safety of the axillo-axillary shunt technique for ensuring continuous ACP during the branch-first stage of total aortic arch replacement in patients with TAAD complicated by CM. As in our first smaller case series, the results of this study show that the off-pump strategy can successfully maintain bihemispheric ACP, minimize neurological complications, and support favorable surgical outcomes [6].

Importance of continuous cerebral perfusion

In cases of TAAD with CM, the risk of irreversible ischemic injury is a major concern during surgery. The conventional use of CPB to support systemic circulation and ACP during these complex reconstructions introduces multiple risks, which can exacerbate neurological deficits [4,5,11]. The axillo-axillary shunt, functioning in an off-pump mode, provides an important alternative that offers uninterrupted, pulsatile ACP throughout the critical stages of the procedure.

In our cohort, ACP was maintained within the normal range of mean pressure of 55 ± 8 mmHg and a flow velocity of 600 ± 90 ml/min, demonstrating the capacity of the shunt to support sufficient cerebral oxygenation. The adequacy of this pressure and flow velocity for cerebral perfusion has been reported by Ganzel et. al. and Suárez et. al. [12,13]. The regional cerebral oxygen rSO2, monitored using cerebral oximetry, remained consistently within the reference range of 60-80%, with no episodes of critical desaturation (below 40%) [14,15]. This highlights the effectiveness of the shunt in providing stable and reliable perfusion, even in patients with significant CM preoperatively.

Comparison to traditional techniques

For the reconstruction of the aortic arch and AAB, several techniques have been proposed. In cases of proximal AAB lesions, multi-branched aortic arch grafts, such as the Gelweave Plexus 3 and Plexus 4, are commonly used. This method involves creating the distal anastomosis with the descending thoracic aorta, followed by stepwise reattachment of the AAB to the graft’s branches [16]. These grafts provide excellent structural support but still require the lengthier use of CPB, which introduces its own risks.

Hybrid techniques, such as the frozen elephant trunk procedure, have also gained wide usage, particularly with the implantation of the Thoraflex Hybrid graft (Terumo Aortic, Inchinnan, Scotland). This method allows for initial ACP through the intact AAB, followed by perfusion through the graft branches and eventually through the implanted aortic graft after the completion of both proximal and distal anastomoses. Additional selective ACP can be achieved with the use of a separate head pump [17,18]. M. Shrestha and colleagues, based on their experience with the Thoraflex Hybrid prosthesis in 100 patients with aneurysms and aortic arch dissection, reported a mortality rate of 7% (versus 5.6% in this study) and stroke occurrence in 9% (versus 0% in this study) of cases [19]. While hybrid techniques like these have been shown to provide adequate cerebral and systemic perfusion, they still involve some of the challenges associated with CPB, including periods of interrupted ACP [6]. Furthermore, the introduction of debranching techniques and improved graft designs, such as the E-vita graft (Artivion, Inc., Kennesaw, Georgia, US), holds promise for enhancing the safety and efficiency of aortic arch reconstruction [20,21]

However, the axillo-axillary shunt technique provides uninterrupted bihemispheric ACP without the need for CPB during the branch-first stage, thus minimizing the risk of embolic events and cerebral ischemia [6]. Additionally, the perfusion circuit can be switched to on-pump mode if necessary, offering flexibility during the procedure (no such instance occurred in this study). This combination of uninterrupted ACP and flexibility makes the axillo-axillary shunt a valuable alternative, particularly in high-risk patients [6].

Neurological outcomes

One of the most significant findings of this study is the absence of new permanent neurological deficits or strokes, which is significantly lower than stroke rates of upto 11% reported in other studies [22,23]. Postoperative assessments revealed that 3 (16.7%) patients experienced transient neurological symptoms, including confusion and transient ischemic attacks (TIAs), which resolved within 48 hours; this is less than the rate of such complications reported in other studies [24,25]. These favorable neurological outcomes may be attributed to the uninterrupted bihemispheric perfusion provided by the shunt, as well as the close intraoperative monitoring of cerebral oxygenation and perfusion using cerebral oximetry and transcranial Doppler.

In addition to preventing ischemic brain injury, the stable rSO2 values observed throughout surgery suggest that the axillo-axillary shunt is effective in preserving cerebral autoregulation, even in patients with pre-existing CM. The avoidance of any critical rSO2 desaturation below 40% further confirms the safety of this technique in maintaining adequate oxygen delivery to the brain [16,17].

Surgical and postoperative outcomes

The overall surgical success rate was high, with all patients undergoing successful branch-first total aortic arch reconstruction using the trifurcation graft. The mean CPB time of 145.3 ± 48.6 minutes and cross-clamp time of 100.6 ± 17.4 minutes were better than the times (227 ± 32 minutes and 147 ± 23 minutes, respectively) reported in other studies on reconstruction procedures for TAAD [22,26]. Importantly, the early postoperative period was free of significant complications, including major bleeding, graft infection, or the need for reoperation. All patients were discharged approximately two weeks after surgery, demonstrating that the axillo-axillary shunt technique not only supports favorable neurological outcomes but also contributes to overall surgical success.

Follow-up CT angiography confirmed that all patients had well-reconstructed and patent aortic arches and branches, with no evidence of CM or graft-related complications. These findings further underscore the reliability and safety of this adaptive perfusion strategy in high-risk aortic surgeries.

Limitations and future directions

While this study demonstrates the effectiveness of the axillo-axillary shunt technique, some limitations should be noted. First, the sample size was relatively small, which limits the generalizability of the findings and reduces the statistical power of the study and the ability to detect rarer complications. Moreover, the small cohort size may not fully represent a diverse patient population; therefore, larger multicenter studies are needed to further validate the results and compare the outcomes of this technique to other perfusion strategies. Additionally, long-term follow-up is required to assess the durability of the grafts and the potential for late neurological or graft-related complications. Abnormal anatomic variations of the AAB, such as bovine arch, might make this technique impractical.

Future research should also explore the application of this off-pump technique in other aortic pathologies, such as aneurysms or dissections involving more distal portions of the aorta. Further refinements in monitoring and perfusion strategies may enhance the safety and efficacy of this approach, particularly in patients with more complex vascular anatomies or comorbid conditions.

## Conclusions

The axillo-axillary shunt technique demonstrated in this study offers a safe and effective approach for maintaining uninterrupted bihemispheric cerebral perfusion during the branch-first stage of aortic arch replacement in patients with Type A aortic dissection complicated by cerebral malperfusion. By eliminating the need for cardiopulmonary bypass during critical stages of the procedure, this technique minimizes the risks (including neurological complications and mortality) associated with lengthier hypothermia, non-pulsatile blood flow, and interrupted cerebral perfusion, which are typically seen with on-pump strategies. Our results show that the axillo-axillary shunt effectively preserves cerebral autoregulation, reduces the incidence of neurological complications, and contributes to favorable surgical outcomes. Despite the advancements in the development of branched aortic arch grafts and hybrid techniques like the frozen elephant trunk procedure, challenges remain, particularly related to the size and positioning of graft branches and the need for CPB. The axillo-axillary shunt technique addresses these challenges by ensuring uninterrupted cerebral perfusion without the limitations of on-pump systems. Expected long-term benefits of this novel technique include better survival, freedom from reoperation, quality of life, and freedom from neurological events. However, larger studies are needed to further validate the long-term benefits of the axillo-axillary shunt, as well as its potential integration with hybrid approaches. Continued refinement of perfusion strategies and monitoring techniques will be essential to further reduce neurological risks and optimize outcomes in high-risk patients undergoing complex aortic surgeries.

In conclusion, the axillo-axillary shunt represents a valuable advancement in the management of aortic arch reconstruction, particularly in cases where cerebral malperfusion poses a significant threat. Its ability to provide continuous cerebral perfusion, maintain stable hemodynamics, and reduce reliance on CPB makes it a promising option for future aortic arch surgeries.
